# New tricks with old dogs: personalised medicine and clinical trials

**DOI:** 10.1038/bjc.2011.299

**Published:** 2011-08-23

**Authors:** J A Koziol

**Affiliations:** 1 Department of Molecular and Experimental Medicine, MEM280, The Scripps Research Institute, 10550 North Torrey Pines Road, La Jolla, CA 92037, USA

**Keywords:** Phase 2 trials, nomograms, Bayesian trials

## Abstract

We provide a didactic example of how clinical trials can accommodate individualised patient information relative to design and analysis.

Do old paradigms remain relevant in this era of personalised medicine? Oncologists often design early Phase 2 trials as single arm studies, with dichotomous clinical outcomes as primary efficacy endpoints. There are hypothesised population values for the target endpoints of interest; and, comparison of observed outcomes from the trial with these population values are then utilised to justify further clinical testing. In this commentary, we argue that one might improve on the design and analysis of such trials through the use of individualised information.

We begin with a motivating example. The author recently consulted on a clinical study aimed at assessing the efficacy of adjuvant multimodality therapy in patients at high risk for prostate cancer recurrence after radical prostatectomy (Michael Lilly, University of California Irvine Comprehensive Cancer Center, personal communication). A single arm Phase 2 study was conducted, with biochemical recurrence constituting the primary efficacy endpoint. It was hypothesised that 2-year non-recurrence exceeding 90% would warrant further clinical investigation of the new therapy. Twenty-four patients were initially enrolled, and two recurrences were observed within 2 years of prostatectomy. Should the trialists be encouraged by the seemingly positive outcome of this trial?

The 90% target represents a global assessment, and represents the trialists’ prior judgment of a clinically significant outcome (Adjei *et al*, 2009). Nevertheless, this target outcome can be refined with individualised information from the study patients. For example, such individualised information is available from nomograms, which present tailored individual predictions of clinical outcomes based on patient characteristics known to be predictive of the outcome of interest. Several validated nomograms for disease recurrence after radical prostatectomy for prostate cancer have been developed (Feller, 1968; Kattan *et al*, 1998, 1999; Berry, 2006). In particular, these nomograms have been shown to predict actual clinical outcomes with high accuracy. We will illustrate how these nomogram assessments can be used as a comparator in our clinical trial, with emphasis on whether observed disease recurrence differs from what might be expected with nomogram prediction. Readers interested in the mathematical details can refer to the appendix; here, we summarise the main finding: if the nomogram probabilities are assumed to be accurate and well calibrated, and if the subjects enrolled in the trial have similar attributes to the training population used for nomogram development, then the probability of observing two or fewer failures by 2 years is less than one in fifty if adjuvant treatment is merely equivalent to standard of care.

We believe the use of individual estimates as comparators in the clinical trial setting is more appropriate than a global target, so long as the individual estimates are well calibrated, that is, that actual outcomes are accurately predicted by the estimated outcome probabilities. Perhaps a less contentious use of nomogram estimates in this setting relates to patient selection: one might hope to improve patient homogeneity, or the possibility of discerning treatment efficacy, by restricting entry to patients at perceived higher risk of progression. These patients would be more appropriate candidates for intensive therapy, such as adjuvant therapy administered after radical prostatectomy, than patients with a low *a priori* likelihood of disease progression. As a reviewer has commented, this notion of enriching a clinical trial with likely responders is very appealing, and should lead to more efficient trials. See Roach *et al* (2006) for related discussion.

We chose a validated nomogram for prediction of biochemical recurrence following radical prostatectomy. As a reviewer has commented, there are a plethora of available nomograms, and some discernment is needed when selecting one for comparator purposes. The nomogram we have selected aligns with the inclusion/exclusion criteria of our particular trial; and, importantly, it has been shown to be well calibrated. Hence the individualised predictions arising from the nomogram-derived probabilities should constitute an improvement over a global assumption that recurrence would occur at a fixed rate in the study cohort (as would be assumed in a ‘standard’ Phase 2 trial). Although perfect prediction would be ideal, reasonably high predictive accuracy is a realistic goal.

It has been argued (Shariat *et al*, 2008) that nomograms are the best available predictive tools for clinical outcomes (in terms of accuracy and discriminating characteristics) in prostate cancer. Nevertheless, alternatives to nomograms as comparators can be devised. The Stephenson nomogram is based on a Cox proportional hazards regression model, and the use of such a regression model would be another option for generating individualised predictions. Or, one could construct a more ‘modern’ nomogram, by incorporating molecular marker information or other potential predictors into the underlying algorithm. The issue then becomes, whether predictive accuracy is enhanced with these modern nomograms, relative to the available standards.

Intrinsic patient heterogeneity in clinical trials impacts both design and analysis. Suppose, for example, we were to design a Phase 2 trial to achieve a specified precision in the estimated outcome probability, based on the assumption that the clinical outcomes will be binomially distributed. If we fail to incorporate the variability in expected responses between patients (overdispersion in the responses relative to binomial variability), our design will be underpowered. Bayesian clinical trials Roach *et al* (2006) provide a natural framework for accommodating overdispersion in response distributions resulting from patient heterogeneity, and should become increasingly prominent in this era of personalised medicine.

## Figures and Tables

**Figure 1:**
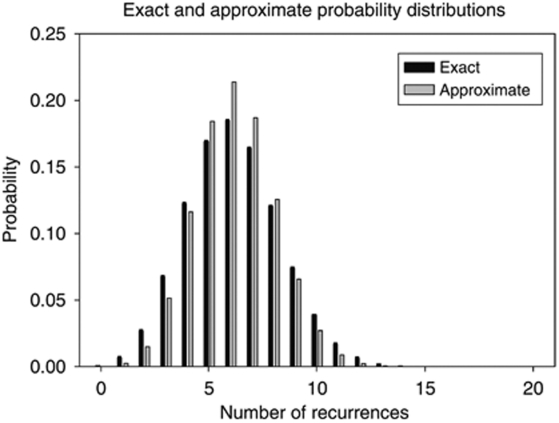
Exact and approximate probability distributions of numbers of recurrences at 2 years. The exact probability distribution is obtained from the individualised nomogram-derived probabilities of recurrence for the 24 patients, as detailed in the Appendix. The approximate probability distribution is a binomial distribution, with *n*=24, and recurrence probability 0.26 (the mean of the 24 individual probabilities, from Table 1).

**Table 1:** Clinical outcomes of 24 patients enrolled in a clinical trial assessing the efficacy of adjuvant multimodality therapy in patients at high risk for prostate cancer recurrence after radical prostatectomy

**Recurrence at 2 years**	**Estimated probability of non-recurrence at 2 years**	**Estimated probability of recurrence at 2 years**
N	0.99	0.01
N	0.98	0.02
N	0.96	0.04
N	0.95	0.05
N	0.94	0.06
N	0.92	0.08
N	0.91	0.09
N	0.91	0.09
N	0.90	0.10
N	0.87	0.13
N	0.85	0.15
N	0.82	0.18
N	0.81	0.19
N	0.75	0.25
N	0.69	0.31
N	0.69	0.31
N	0.63	0.37
N	0.55	0.45
N	0.52	0.48
N	0.52	0.48
N	0.40	0.60
N	0.21	0.79
Y	0.76	0.24
Y	0.31	0.69

Abbreviations: N=no progression; Y=disease progression.

The estimated 2-year recurrence probabilities are nomogram based (Stephenson *et al.*, 2005). The recurrence probabilities are denoted as *p*_i_ in the Appendix, with *q*_i_=1–*p*_i_.

## APPENDIX

We initially accessed the Memorial Sloan Kettering on-line prostate cancer nomogram from Stephenson *et al* (2005) (http://www.mskcc.org/applicat ions/nomograms/prostate/PostRadicalProstatectomy.aspx), which predicts the probability of freedom from biochemical recurrence of disease (failure) within 2 years following prostatectomy, based on known risk factors pre-treatment PSA level, Gleason grade and pathologic features of the prostatectomy specimen. In Table 1 we list the clinical outcomes of the 24 patients, along with these nomogram-based probabilities of freedom from disease recurrence by 2 years.

Is the observed number of failures (2 out of 24) significantly smaller than would be expected from the nomogram predictions? Comparison of observed clinical outcomes with the nomogram predictions can be effected in the following manner. Under the null hypothesis that treatment outcomes are no better than one would expect from prostatectomy alone, as reflected by the nomogram assessments, the individual outcomes can be taken as independent Bernoulli random variables, with probabilities of failure derived from the nomogram calculations (A Bernoulli random variable is a discrete random variable assuming one or another of two states, with associated probabilities summing to one. The outcome of a random coin flip is a canonical example of a Bernoulli random variable: note that the coin need not be unbiased!) In particular, these Bernoulli variables are not necessarily identically distributed, as their respective failure probabilities may be different. Hence the total number of observed treatment failures will not in general have a simple binomial distribution; rather, its distribution is a convolution of independent, non-identically distributed random variables. This distribution can be computed exactly, using probability generating functions. The theory of probability generating functions for discrete random variables is well known; see, for example, Feller’s classic text (Feller, 1968) for an enlightening introduction. Here we will merely cite salient results relevant to the problem at hand.

Formally, let p_i_ denote the nomogram-based probability of disease recurrence by 2 years for the ith patient, *i*=1, 2,…, 24. Let X_i_ denote the binary random variable, *X*_i_=1 if the ith patient experiences recurrence, 0 otherwise; its distribution is simply Pr(*X*_i_=1)=*p*_i_, Pr(*X*_i_=0)=*q*_i_=1−*p*_i_. Then the probability generating function (pgf) *G*_i_(*s*) of *X*_i_ is given by *G*_i_(*s*)=(*q*_i_+*p*_i_**s*); and, the pgf of  is given by the product

The exact probability distribution of S_24_ is easily obtained from *G*(*s*): Pr(*S*_24_=*j*) is merely the coefficient of *s*^*j*^ in the power series expansion of *G*(*s*), *j*=0,1,…,24. We depict this distribution in Figure 1. We observed *S*_24_=2 recurrences (failures); the corresponding one-sided exact *P*-value consists of the probabilities of 2 or fewer failures from the null distribution in Figure 1 is Pr(*S*_24_=0)+Pr(*S*_24_=1)+Pr(*S*_24_=2)=0.000185+0.002532+0.014973=0.0177 (We note in passing that the cumulative distribution function of S_24_ can also be obtained by generating functions, so tail probabilities can themselves be calculated without recourse to summation). We utilised Mathematica 6.0 (Wolfram Research, Inc., Champaign, IL, USA) for the calculations detailed here, as Mathematica provides exceptional capabilities for symbolic arithmetic; but other programs are readily available. Alternatively, one can compute the relevant probabilities from first principles: a simple example is given in the Excursus.

As a basis for comparison, we also include in Figure 1 an approximation to the exact distribution of the number of recurrences. The approximation is based on the binomial distribution, derived as follows. From Table 1, the mean probability of recurrence is 0.26 (this is the average of the 24 individual probabilities in the last column). The approximate probability distribution depicted in Figure 1 is merely a binomial distribution, with parameters *n*=24, and *P*=0.26. The exact distribution is overdispersed relative to the binomial distribution: variability in the exact distribution is larger than in the binomial distribution. This overdispersion affects calculation of tail probabilities: for example, the probability of two or fewer recurrences in 24 subjects, each with recurrence probability 0.26, is 0.0034. Neglecting inter-subject variability in likelihood of recurrence results in an overly optimistic assessment of statistical significance.

### Excursus

We provide a simple example of the calculations detailed in the Appendix. Suppose we have three patients, with probabilities of disease recurrence *p*_i_, *i*=1,2,3, respectively, and *q*_i_=*1*−*p*_i_; then, it is straightforward to calculate the following summary probabilities:

Exact enumeration of probabilities of observed numbers of recurrences is feasible with larger sample sizes, but at a cost of increased bookkeeping complexity. In comparison, the probability generating function approach detailed in the Appendix entails calculation of

It is immediately apparent that the coefficient of *s*^j^, *j*=0,1,2,3, corresponds to the probability of observing *j* recurrences, as in the above Table. The advantage of symbolic calculation of the probability generating function will become increasingly appreciated as the sample size increases.
